# Arthroscopic Bone Marrow Stimulation for Non-primary Osteochondral Lesions of the Talus Yields Limited Improvements in Patient-Reported Outcomes Compared to Primary Lesions: A Prospective 2-Year Follow-up Study

**DOI:** 10.1177/10711007251405240

**Published:** 2026-02-24

**Authors:** Quinten G. H. Rikken, Jari Dahmen, Julian J. Hollander, Jason A. H. Steman, Sjoerd A. S. Stufkens, Gino M. M. J. Kerkhoffs

**Affiliations:** 1 Department of Orthopedic Surgery, Amsterdam Movement Sciences, Amsterdam UMC, Location AMC, University of Amsterdam, the Netherlands; 2 Academic Center for Evidence based Sports medicine (ACES), Amsterdam UMC, the Netherlands; 3 Amsterdam Collaboration for Health and Safety in Sports (ACHSS), International Olympic Committee (IOC) Research Center, Amsterdam UMC, the Netherlands

**Keywords:** osteochondral lesion talus, OLT, bone marrow stimulation, BMS, non-primary

## Abstract

**Background:**

Our aim in this study was to prospectively assess the patient-reported clinical outcomes of arthroscopic bone marrow stimulation (BMS) for non-primary osteochondral lesions of the talus (OLT) and to compare these with primary cases at 2-year follow-up. The secondary aims were to assess the association of baseline factors with outcomes and the occurrence of adverse events.

**Methods:**

Patients who underwent arthroscopic BMS were prospectively included and assessed up to 2-year follow-up and were grouped according to non-primary (ie, failed previous OLT surgery) or primary BMS. Patient-reported outcomes were collected at baseline and 2-year follow-up and included the numeric rating scale (NRS) for pain and the Foot and Ankle Outcome Score (FAOS) questionnaires. The primary outcome was the improvement in NRS pain during walking, with a minimal clinically important difference (MCID) of 2.0. Adverse events concerned reoperations and complications during the study period.

**Results:**

Forty-four patients were included: 25 in the primary group and 19 in the non-primary group. Both groups showed a statistically significant improvement in pain and functional outcomes from preoperatively to 2-year follow-up. The improvement in the primary outcome was significantly higher in the primary group (median 3 [IQR: 1-5] out of 10) compared to the non-primary group (median 1 [IQR: 1-3] out of 10), *P* = .01. Moreover, 68% (95% CI 46%-85%) reached the MCID compared with 32% (95% CI 13%-57%) in the non-primary group, which was statistically significant (*P* = .03). Baseline variables showed no consistent association with the primary outcome, except for a moderate correlation with age and lower improvements for patients who received concomitant surgery in the primary group. None of the changes in the FAOS sub-scales showed a statistically significant difference between the two groups. Two revision procedures (non-primary group: 11% [95% CI 1%-33%] vs 0% primary group, *P* = .2) occurred in the non-primary group. During the study period, 1 case (non-primary group: 5% [1%-26%] vs primary group: 0%, *P* = .4) had a complication.

**Conclusion:**

The most important finding of this prospective study is that arthroscopic BMS for non-primary OLT yields a significant improvement in patient-reported outcomes compared with baseline, but an inferior improvement compared with primary OLT at 2-year follow-up. On average, approximately two-thirds of BMS-treated primary OLTs reached the MCID compared with one-third in the non-primary group.

**Level of Evidence:**

Level II, prospective comparative cohort study.

## Graphical abstract

**Graphical Abstract:**
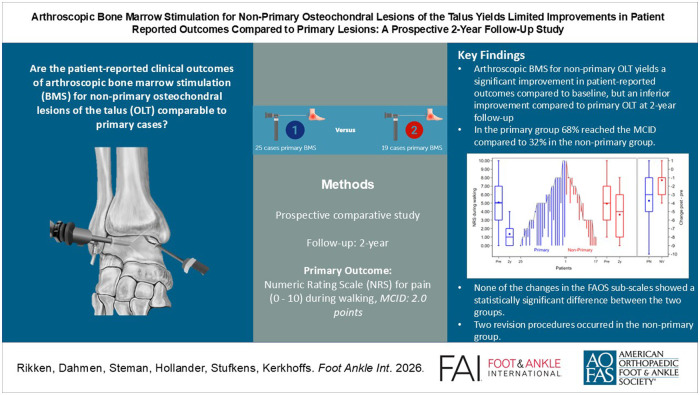
This is a visual representation of the abstract.

## Introduction

Arthroscopic bone marrow stimulation (BMS) is the most common surgical treatment for small (<150 mm^2^), primary, osteochondral lesion of the talus (OLT).^1^ Its advantages over other surgical options are its relatively less invasive nature, faster return to weightbearing and sporting activities, lower costs, no donor site morbidity, and technical simplicity.^2-4^ The clinical results of arthroscopic BMS up to mid-term follow-up are good and appear satisfactory at long-term follow-up.^1,5,6^ Despite these positive results, a number of controversies surrounding BMS remain.

One such controversy is the use of BMS in non-primary OLT (ie, failed prior surgical treatment).^7,8^ Previous studies reported varying results from non-primary BMS, whereas (osteo)chondral replacement or chondrogenesis-inducing techniques have reported good outcomes in non-primary lesions.^9,10^ These techniques can be costly and relatively more invasive, however. Clinically, non-primary BMS may be an option in previously inadequately treated lesions, and patients may benefit from its less invasive nature and lower costs. A recent systematic review on non-primary BMS found only small improvements in patient outcomes; however, the current literature consists of small (retrospective) non-comparative case-series.^7,10-12^ There is, therefore, insufficient data to make an evidence-based statement on the indication and efficacy of arthroscopic BMS for non-primary OLT. Moreover, studying non-primary BMS could identify patients who may benefit from the procedure, thus expanding the treatment options for OLT patients.

The present study, therefore, aimed to prospectively assess the patient-reported clinical outcomes of non-primary BMS for OLT and to compare these with primary BMS cases at 2-year follow-up. The secondary aims were to assess the association of baseline factors with outcomes and the occurrence of adverse events. It was hypothesized that primary BMS would result in superior outcomes compared with non-primary BMS.

## Methods

The present study is a prospective comparative study performed at a tertiary academic referral hospital specialized in the treatment of cartilage lesions in the ankle. The study was approved by the local medical ethics committee (reference number: W14_237#14.17.0288) and is in accordance with the declaration of Helsinki.

### Patient Selection

Patients who underwent arthroscopic BMS (debridement and/or microfracturing) between March 2018 and July 2022 were prospectively assessed and included for the study according to the inclusion and exclusion criteria as reported in Table 1. Patients were eligible for arthroscopic BMS in case of a symptomatic OLT unresponsive to non-operative treatment for a minimum of 3-6 months and counseled for surgery in a shared decision-making process. All patients provided consent before inclusion.

**Table 1.** Inclusion and Exclusion Criteria.

Inclusion Criteria	Exclusion Criteria
Patient who underwent arthroscopic BMS for a symptomatic OLT	Ankle osteoarthritis (>Cohen grade 2) at baseline on CT scan
and	Concomitant osteochondral lesion of the tibial plafond
CT scan before surgical treatment	Systemic disease affecting the ankle; including rheumatoid arthritis and hemophilic arthropathy
	Ankle fracture at baseline
	Not able or willing to participate
	Lost to follow-up

Abbreviations: BMS, bone marrow stimulation; CT, computed tomography; OLT, osteochondral lesion of the talus.

### Data Collection

Baseline demographic and surgical information was extracted from the electronic patient records using a predefined extraction form using CASTOR. Baseline demographics included sex, age, body mass index (BMI), traumatic injury etiology, smoking status, and any prior foot or ankle surgery. Treatment characteristics included arthroscopic approach (anterior, posterior, both), type of BMS (debridement alone or with microfracturing), any concomitant surgical intervention, and postoperative rehabilitation protocol.

### Clinical Outcomes

#### Patient-reported outcome measures

All patient-reported outcome measures (PROMs) were prospectively collected using the online CASTOR portal by a researcher not involved in clinical care.

The primary outcome of this study concerned the improvement of the numeric rating scale (NRS) for pain during walking from baseline to 2-year follow-up. The NRS for pain ranges from 0 (no pain) to 10 (worst imaginable pain). Furthermore, the proportion of patients exceeding the minimal clinically important difference (MCID) of the primary outcome in both groups was calculated. The secondary PROMs concerned the NRS for pain during rest, running, and stairclimbing. Additionally, the foot and ankle outcomes score (FAOS) was collected. The FAOS is a functional outcome score and measures from 0 (lowest) to 100 (highest) and consists of 42 questions distributed among 5 subscales: symptoms, pain, activities of daily living, sport, and quality of life.

#### Adverse events

Any postoperative complication or reintervention was prospectively collected in the electronic patient health records and reviewed. Reoperation was divided into revision surgery (any surgical intervention affecting the OLT) and reoperation for any other reason. Nonsurgical interventions in the postoperative period were reported separately.

### Radiologic Assessment

Computed tomography (CT) scans were available for all patients at baseline and were assessed by 2 independent raters (Q.R. and J.D.) for lesion characteristics. The baseline assessment consisted of lesion size measurements (anterior-posterior direction, medial-lateral direction, and depth), lesion location according to Raikin et al,^13^ and lesion morphology according to Rikken et al.^14^ A pre-operative CT-based evaluation of ankle osteoarthritis grading was conducted according to Cohen et al.^15^

### Statistical Analysis

A power analysis for the primary outcome indicated a minimum sample size of 13 cases was needed to detect an MCID of 2.0 out of 10 points (NRS during walking), assuming an SD of 1.5 using a Wilcoxon rank-sum test with a 2-sided .05 significance level and 80% power (nQuery advisor 8.5.1; Statistical Solutions Ltd).^9,16^ To correct for a potential loss to follow-up of 20%, the required minimum sample size for the present study was 16 cases.

All data analyses were conducted using Stata 17 (StataCorp LP). A 2-sided level of *P* < .05 was considered significant. Baseline characteristics were reported in absolute numbers with percentages for dichotomous and categorical variables, and medians with interquartile ranges (IQRs) for continuous values. Normality of data was visually assessed and with a Shapiro-Wilk test. A Fisher exact test and Mann-Whitney *U* test were used to compare baseline characteristics between the primary and non-primary groups. Patient-reported outcomes, including the primary outcome, were analyzed with a Mann-Whitney *U* test between the primary and non-primary group. A Wilcoxon signed-rank test was used when comparing baseline values to 2-year postoperatively within each group. The difference in proportions of MCID reached (including revision surgery as a failure) per group for the primary outcome, as well as the rate of adverse events, was assessed using a Fisher exact test. A Wilson score method (without continuity correction) was used to calculate 95% CIs for these proportions. To assess the association of covariates with the primary outcome, a Spearman ρ test was used. The Spearman ρ was interpreted according to Schober et al.^17^ Sub-analyses were performed on the primary outcome for non-continuous baseline variables in both groups, using a Fisher exact test for binary variables and Kruskal-Wallis test for ordinal variables >2 groups.

## Results

**Figure 1.**
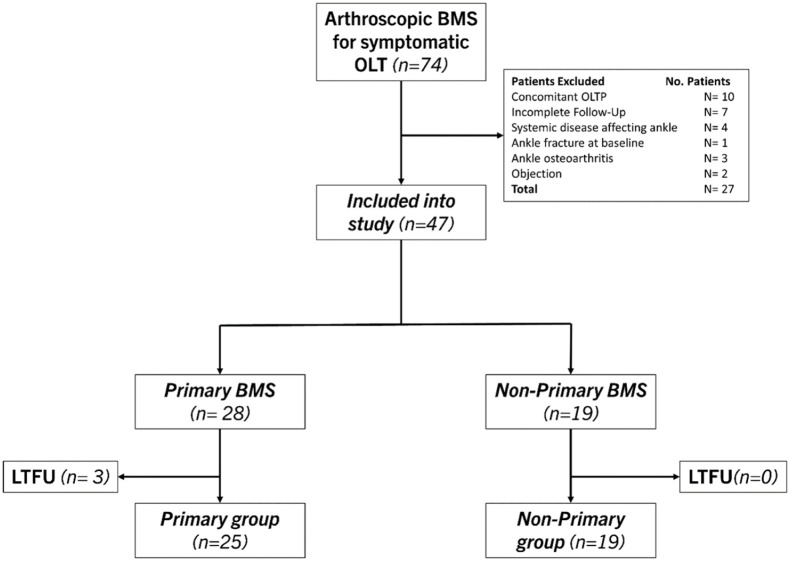
Flowchart of patient selection.

**Figure 2.**
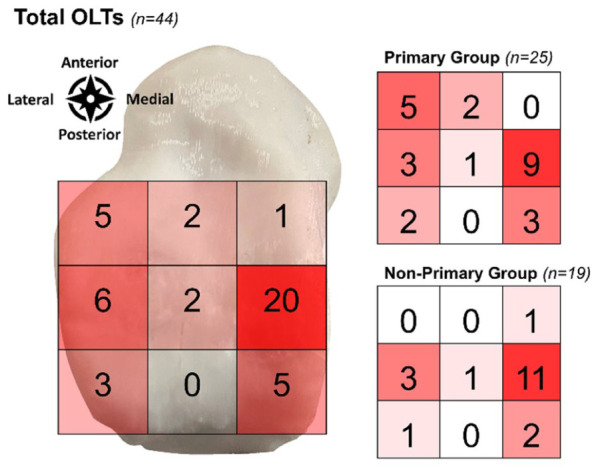
Lesion localization according to a 9-grid scale.

At final follow-up, 47 eligible patients were included in the study (Figure 1). Of these, 44 patients (94%) had a complete follow-up, and 25 patients were included in the primary group and 19 patients in the non-primary group. There were no significant differences in baseline patient and treatment characteristics (Table 2). In terms of lesion characteristics, there was a significant difference in lesion morphology as well as the osteoarthritis grading between the 2 groups; see Table 3. Lesion localization is depicted in Figure 2.

**Table 2.** Baseline Patient and Treatment Characteristics.

Characteristics	Primary Group (n = 25)	% reported	Non-primary Group (n = 19)	% reported	*P* Value
Patient characteristics					
Sex, n (% male)	10 (40)	100	12 (63)	100	.2
Age, y, median (IQR)	25.6 (19.0-36.5)	100	28.0 (23.8-33.6)	100	.3
BMI, median (IQR)	23.8 (21.6-24.5)	100	24.2 (21.2-26.6)	100	.5
Smoking status, n (%)		88		95	.4
Non-smoker	20 (91)		14 (78)		
Active smoker	2 (9)		4 (22)		
Previous ankle trauma, n (%)		96		68	.4
Yes	20 (82)		9 (69)		
No	4 (18)		4 (31)		
Specified (% of total no. trauma)					
Sprain	13 (65)		5 (56)		
Axial impact or fall from height	2 (10)		0		
Direct impact trauma	2 (10)		0		
Fracture	1 (5)		1 (11)		
Mechanism unknown	2 (10)		3 (33)		
Number of previous OLT procedures, n	NA		25	100	NA
Per patient, median (IQR)	NA		1 (1-2)		
Specified in procedures (% of total)					
Arthroscopic BMS			21		
Open BMS			1		
Fixation			1		
Autologous bone grafting			2		
Treatment characteristics					
Surgical approach, n (%)		100		100	.4
Anterior	23 (92)		15 (79)		
Posterior	1 (4)		1 (5)		
Both	1 (4)		3 (16)		
BMS technique, n (%)		100		100	.9
Debridement only	2 (8)		1 (5)		
Debridement with microfracturing	23 (92)		18 (95)		
Concomitant surgery, n (%)	14 (56)	100	10 (53)	100	.9
Total no. of	16		12		
Specified per procedure (% total no. of)					
Impingement removal – bony	6 (39)		7 (58)		
Impingement: soft-tissue	1 (6)		1 (8)		
Removal free body	4 (25)		0		
Duquenoy (open)	1 (6)		1 (8)		
ATFL repair (arthroscopic)	1 (6)		0		
Tightrope syndesmosis	1 (6)		0		
FHL release	1 (6)		1 (8)		
Excision posterior talar process	0		1 (8)		
Removal os trigonum	1 (6)		1 (8)		

Abbreviations: ATFL, anterior talofibular ligament; BMI, body mass index; BMS, bone marrow stimulation; FHL, flexor hallucis longus; NA, not applicable; OLT, osteochondral lesion of the talus.

**Table 3.** Baseline Lesion Characteristics.

Lesion Characteristics	Primary Group (n = 25)	Non-primary Group (n = 19)	*P* Value
Morphology, n (%)			
Cystic	9 (36)	4 (21)	**.03**
Crater	6 (24)	12 (63)	
Fragment	10 (40)	3 (16)	
Size, mm, median (IQR)			
Anterior-posterior	12 (8-15)	12 (9-15)	.7
Medial-lateral	8 (7-11)	8 (7-10)	.4
Depth	6 (5-8)	5 (4-6)	.3
Area, mm^2^	77.4 (50.6-113.8)	78.2 (66.4-123.2)	.4
Pre-operative ankle osteoarthritis stage^15^			
Median (IQR)	1 (0-1)	1 (0-1)	
Stage, n (%)			
Stage 0	10 (40)	2 (11)	**.04**
Stage 1	12 (48)	16 (84)	
Stage 2	3 (12)	1 (5)	
Stage 3	–	–	
Osteophytes present, n (%)	15 (60)	17 (89)	**.04**

Boldface indicates significance (*P* < .05).

### Clinical Outcomes

**Figure 3.**
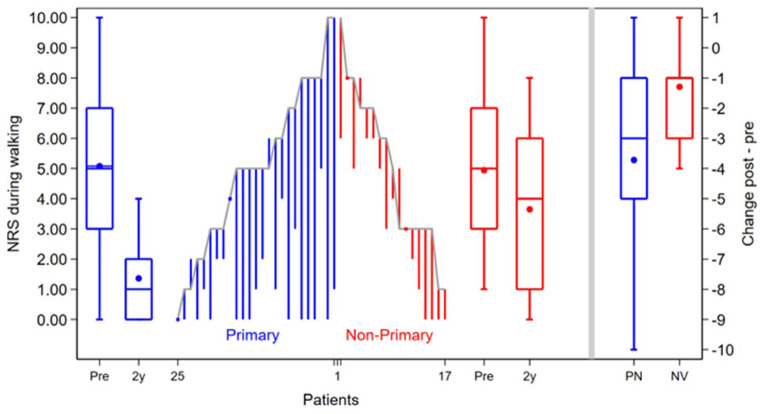
Depiction of the per case change of the NRS during walking from preoperatively to 2-year postoperatively for primary group (blue) and non-primary group (red). Boxplots depict the preoperative (pre) and 2-year postoperative (2y) outcomes on a group level (IQR indicated by a box with line as median value, mean is depicted as a dot in the box, and whiskers depict the absolute values). On the right, the change at a group level from preoperative to 2 years postoperatively is depicted per group. [See online article for color figure.]

The primary outcome, the NRS during walking, significantly improved from baseline to 2 years postoperatively in the primary group (median 5 [IQR: 3-7] to 1 [IQR: 0-2], *P* < .01) and the non-primary group (median 6 [IQR: 3-8] to 4 [IQR: 1-6], *P* < .01). The improvement in the primary outcome was significantly higher in the primary group (median 3 [IQR: 1-5] out of 10) compared with the non-primary group (median 1 [IQR: 1-3] out of 10), *P* = .01) (Figure 3). Moreover, in the primary group, 68% (95% CI 46%-85%) reached the MCID compared with 32% (95% CI 13%-57%) in the non-primary group, which was statistically significant (*P* = .03). In the analysis of association between baseline variables and the primary outcome, it was observed that there was a moderate correlation (ρ = 0.4, *P* = .04) of age with the primary outcome in the primary group (Appendix 1). In the sub-analysis of the primary group, there was a significant difference in the primary outcome for patients who had received concomitant surgery (Appendix 2). There were no other baseline variables associated with the primary outcome in both groups.

With respect to the secondary outcome measures, there was a statistically significant difference in the improvement of the NRS during stairclimbing, with the primary group (median: 4 [IQR: 1.5-7.5]) showing a higher improvement than the non-primary group (median 1.5 [IQR: −0.5 to 3.5], *P* = .04). None of the changes in the FAOS sub-scales from pre-operatively to 2 years postoperatively showed a statistically significant difference between the primary and non-primary groups. An overview of all PROMs is available in Table 4.

**Table 4.** Comparison of Clinical Outcomes for the Primary and Non-primary BMS Group.^a^

	Primary Group (n = 25)				Non-primary Group (n = 19)				Between Groups^b^
	Preoperative	2-y Postoperatively	Improvement	*P* Value	Preoperative	2-y Postoperatively^c^	Improvement^c^	*P* Value	*P* Value
NRS, median (IQR)									
Pain during walking	5 (3 to 7)	1 (0 to 2)	3 (1 to 5)	**<.01**	6 (3 to 8)	4 (1 to 6)	1 (1 to 3)	**<.01**	**.01**
Pain at rest	2 (1 to 4)	0 (0 to 1)	2 (0 to 4)	**<.01**	3 (2 to 5)	2 (0 to 3)	1 (0 to 3)	**<.01**	.4
Pain during running	7 (5.5 to 9.5) (n = 24)	3 (0 to 5.5) (n = 24)	3 (2 to 6) (n = 23)	**<.01**	7 (5 to 10) (n = 15)	5 (2 to 10)	2 (0 to 5) (n = 14)	**<.01**	.2
Pain during stairclimbing	5.5 (3 to 8) (n = 21)	0.5 (0 to 3) (n = 24)	4 (1.5 to 7.5) (n = 20)	**<.01**	5.5 (3 to 7) (n = 16)	3.5 (0 to 6) (n = 14)	1.5 (−0.5 to 3.5) (n = 12)	.1	**.04**
FAOS, median (IQR)									
Symptoms	63 (44 to 79) (n = 24)	68 (54 to 79) (n = 23)	4 (−10 to 29) (n = 23)	.1	46 (43 to 61)	54 (39 to 64)	4 (−7 to 11) (n = 17)	.6	.6
Pain	63 (43 to 71) (n = 24)	83 (69 to 94) (n = 23)	22 (6 to 36) (n = 23)	**<.01**	52 (42 to 69)	67 (56 to 89)	11 (3 to 19) (n = 17)	**.03**	.1
ADL	98 (97 to 100) (n = 24)	100 (100 to 100) (n = 23)	0 (0 to 2) (n = 23)	**<.01**	98 (97 to 98)	98 (98 to 100)	0 (0 to 0) (n = 17)	.4	.1
Sport	45 (30 to 55) (n = 24)	65 (55 to 95) (n = 23)	25 (10 to 50) (n = 23)	**<.01**	30 (15 to 45)	45 (35 to 60)	10 (0 to 30) (n = 17)	**<.01**	.2
QOL	31 (19 to 41) (n = 24)	53 (38 to 75) (n = 22)	19 (13 to 31) (n = 22)	**<.01**	31 (19 to 44) (n = 18)	38 (25 to 69)	6 (0 to 34) (n = 16)	**.04**	.2

Abbreviations: ADL, activities of daily living; AOFAS, American Orthopaedic Foot & Ankle Society score; FAOS, Foot and Ankle Outcome Score; NRS, numeric rating scale; QOL, quality of life.

a Boldface indicates statistical significance (*P* < .05).

b Comparison of postoperative outcomes between primary and secondary group.

c Two patients underwent reoperation and were thus not included, otherwise a complete sample is available.

### Adverse Events

During the study period a total of 3 reoperations occurred. From these 3 reoperations, 2 revision procedures (non-primary group: 11% [95% CI 1%-33%] and 0% primary group, *P* = .2) occurred in the non-primary group. From these 2 revision surgeries, 1 case underwent an autologous osteoperiostic grafting from the iliac crest (TOPIC) procedure and 1 case had an autologous bone grafting procedure. There was 1 reoperation (nonprimary group 0% and primary group: 4% [1%-20%], *P* = .9) not pertaining to the OLT, which was the placement of an additional tightrope for symptomatic residual instability of the syndesmosis.

In the study follow-up period, 1 case (non-primary group: 5% [1%-26%] and primary group: 0%, *P* = .4) had a complication. This patient presented 2 weeks postoperatively with a blister from the pressure bandage, which resolved uneventfully. Second, this patient had persistent pain from the saphenous nerve, for which a multi-disciplinary pain treatment was undertaken, and the patient underwent bi-annual pulsed radiofrequency treatment to alleviate symptoms. All other non-surgically related adverse events were reported in Appendix 3 in detail.

## Discussion

The most important finding of this study is that non-primary BMS for OLT yields a significant improvement in patient-reported outcomes compared with baseline, but a smaller improvement compared with primary BMS at 2-year follow-up. On average, approximately two-thirds of BMS-treated primary OLTs reached the MCID compared with one-third in the non-primary group. Revision procedures were infrequent in both groups; within the limitations of the sample size, we did not detect a clear difference in revision rates between primary and non-primary BMS.

### Clinical Outcomes of Non-primary BMS

The use of BMS for non-primary OLT is a topic of debate.^7,8,18^ Recent systematic reviews have shown that BMS for non-primary OLT likely results in inferior clinical outcomes.^7,18^ A limitation outlined in these studies is the level of evidence of these studies, which mostly consist of retrospective non-comparative case series.^7,18^ The largest study on non-primary BMS is the retrospective study by Yoon et al,^9^ which compared clinical outcomes of 22 ankles that underwent non-primary BMS with 22 ankles that underwent autologous osteochondral transplantation (AOT) at 4.2-year follow-up. The authors of the aforementioned study reported significant improvements in pain (visual analog scale) and functional (AOFAS) outcomes up to 1-year follow-up, but reported a deterioration of the results to baseline-level outcomes at final follow-up. These results do not represent the findings of the present study. This difference may be explained by the inclusion of 32% large (>150 mm^2^) lesions in the study by Yoon et al,^9^ which all required revision surgery, and only 41% of cases being treated with microfracturing. It could be hypothesized that the reasons for the reported limited outcomes following non-primary BMS are insufficient fibrocartilage formation or progressive degeneration, subchondral bone damage, and inherent (yet unknown) patient or lesion factors that would preclude a successful result regardless of primary or non-primary BMS. Moreover, another reason could be that patients with pre-existing degenerative changes could benefit less from non-primary arthroscopic BMS. In this study, the classification of pre-operative degenerative changes was not associated with lower PROM in the non-primary group, though these patients overall had a higher grade compared to the primary group. Current long-term literature does not support the progression to end-stage osteoarthritis following BMS in the majority of patients.^6^ However, the grade of degenerative changes could be a predictive factor for the success of repeat BMS and should be studied further.

When directly comparing the outcomes of non-primary BMS to other surgical options for non-primary OLT, such as replacement or regenerative therapies, it is known from the limited literature that non-primary BMS may result in inferior outcomes.^9,10^ However, the associated morbidity, rehabilitation time, and costs of replacement and regenerative therapies should be taken into account when considering the surgical options in non-primary OLT.^3,9,19^ As such, non-primary BMS may be a beneficial treatment option in select cases. First, non-primary BMS could reasonably be considered for small (<150-mm^2^) OLT in patients who do not wish, or are not able, to undergo more invasive surgery (such as an autograft or allograft procedure), in health care systems where alternative options are not (financially) available, in cases of inadequate previous BMS, or in cases where other treatment options are contra-indicated. Second, physicians should incorporate the prognostic lesion and patient characteristics currently known in the literature (eg, lesion size and location, the presence of cysts, smoking status, alignment, instability, and BMI) in their algorithm in order to evaluate the indication for non-primary BMS on an individual basis.^8,20-26^ As such, the indication for non-primary BMS can be embedded in a patient-centered shared-decision treatment algorithm. Further prospective comparative studies with homogenous patient groups should evaluate the efficacy and longevity of non-primary BMS compared to replacement or regenerative treatment options. Clinically, a non-primary BMS procedure is considered if it is paramount for physicians to clearly inform patients on the expected outcomes, where one could reasonably state that there is a limited improvement in pain and functional outcomes following BMS for a non-primary OLT based on the current literature.^7,18^

Another important aspect of the interpretation of the PROMs in the present study is whether patients sufficiently achieved the MCID. Although no formal MCID is available for the NRS (or visual analog scale) in OLT patients, one could state that an MCID of 2 points on the NRS coincides with a “much better” improvement in pain.^16^ When critically examining the results of this study, this change was observed in the majority of primary cases but not in the non-primary cases. A number of non-primary patients may be able to achieve this MCID, although limited evidence is available on prognostic factors that could predict achieving this threshold. Moreover, changing patient expectations and mental health outcomes could also affect this threshold. The expected outcomes for a patient could reasonably change within the setting of a “simple” primary procedure compared to a recurrent OLT, where pain catastrophizing and patients’ sports and/or work demands may change. This could also be a source of bias when comparing primary to non-primary cases.

### Importance of Baseline Factors

One of the aims of this study was to investigate the association of baseline factors with clinical outcomes. Except for a moderate correlation between age and the primary outcome, and concomitant surgery showing better improvements, in the primary group it was found that no baseline patient and lesion factors were correlated. The external validity of these findings is likely low because of underpowering and the findings possibly being due to statistical chance. To date, there is no clear correlation between age and outcomes of BMS for OLT reported in the literature nor a clinically relevant cutoff.^8,27^

From the literature as a whole, it is reported by Yoon et al^9^ that there is a significant association between baseline lesion size and postoperative clinical outcomes. This is in alignment with numerous prior studies on BMS in primary OLT, establishing a relationship between lesion size and postoperative outcomes.^21,28^ Another finding of interest is that we did not observe an association between the number of previous OLT surgeries and the amount of improvement in pain outcomes. The authors hypothesize that there may be a critical threshold for the number of repeat BMS procedures for it to be clinically effective, and that these deteriorate over the number of subsequent procedures. An important note for the observed influence of baseline factors in this study, and the literature in general, is that there is likely underpowering of studies for the assessment of such factors.^7^ Future efforts should, therefore, include larger sample sizes and possibly include (inter)national collaborative efforts to better identify patients who may benefit from repeat BMS.

### Adverse Outcomes

The present study assessed the 2-year revision rate and observed that 2 cases (11%) in the non-primary group required revision surgery. Arshad et al.^7^ reported a 34% (26 of 77 cases reported) revision rate in their systematic review of non-primary BMS at a weighted average follow-up of 52 months (range of means: 12-154). The studies of Chuckpaiwong et al^22^ and Yoon et al^9^ largely (21 of 26 revision cases) contributed to this revision rate, which may be due to the prognostically poorer lesion characteristics included in both studies. In a recent study by Rikken et al,^20^ which investigated the 10-year revision rate in 262 BMS cases (19% non-primary lesions), no increased revision risk for non-primary OLT was observed. In general, caution is warranted with the interpretation of the findings of this study and the present literature because of the relatively short follow-up and risk of bias, considering the variation in patient characteristics and study designs, respectively. Further evaluating the mid-term to long-term revision risk in non-primary OLT cases may assist in assessing its safety and clinical usefulness.

When assessing the complications, it was observed that 1 case had a nerve injury with persistent complaints. Nerve injuries are one of the most common complications following ankle arthroscopy and are often transient.^3,7^ Additionally, 1 case required a reoperation not pertaining to the OLT. In general, it could be stated that in terms of safety, non-primary BMS is not inferior to primary BMS.^7^

### Methodologic Considerations

This study has several strengths and limitations. First, it is a prospective comparative study that includes comparable patient groups. Second, a prospective sample size calculation was conducted for the primary outcome measure. Third, the lesion size measurements were conducted by 2 independent reviewers.

The limitations of this study are the loss to follow-up of 3 cases (6%), possibly including bias and a number of incomplete questionnaires, limiting the power of the analysis for the non-primary outcome measures. Second, the results of the secondary outcome measures and sub-analysis should be interpreted with caution, as the present study included a relatively low number of patients and may therefore be underpowered for these outcome measures. Third, there were several heterogeneities in the patient baseline factors, as well as varying concomitant surgical procedures that could have affected the outcomes. Fourth, the present study did not include an imaging analysis at follow-up.

## Conclusion

The most important finding of this study is that arthroscopic BMS for non-primary OLT yields a significant improvement in patient-reported outcomes compared to baseline, but an inferior improvement compared to primary OLT at 2-year follow-up. Approximately one-third of BMS-treated primary OLTs reached the MCID compared with two-thirds in the non-primary group. The indication for non-primary BMS can be embedded in a patient-centered shared-decision treatment algorithm, where it is paramount for physicians to clearly inform patients on the expected outcomes.

## Supplemental Material

sj-docx-1-fai-10.1177_10711007251405240 – Supplemental material for Arthroscopic Bone Marrow Stimulation for Non-primary Osteochondral Lesions of the Talus Yields Limited Improvements in Patient-Reported Outcomes Compared to Primary Lesions: A Prospective 2-Year Follow-up Study Supplemental material, sj-docx-1-fai-10.1177_10711007251405240 for Arthroscopic Bone Marrow Stimulation for Non-primary Osteochondral Lesions of the Talus Yields Limited Improvements in Patient-Reported Outcomes Compared to Primary Lesions: A Prospective 2-Year Follow-up Study by Quinten G. H. Rikken, Jari Dahmen, Julian J. Hollander, Jason A. H. Steman, Sjoerd A. S. Stufkens and Gino M. M. J. Kerkhoffs in Foot & Ankle International

sj-docx-2-fai-10.1177_10711007251405240 – Supplemental material for Arthroscopic Bone Marrow Stimulation for Non-primary Osteochondral Lesions of the Talus Yields Limited Improvements in Patient-Reported Outcomes Compared to Primary Lesions: A Prospective 2-Year Follow-up Study Supplemental material, sj-docx-2-fai-10.1177_10711007251405240 for Arthroscopic Bone Marrow Stimulation for Non-primary Osteochondral Lesions of the Talus Yields Limited Improvements in Patient-Reported Outcomes Compared to Primary Lesions: A Prospective 2-Year Follow-up Study by Quinten G. H. Rikken, Jari Dahmen, Julian J. Hollander, Jason A. H. Steman, Sjoerd A. S. Stufkens and Gino M. M. J. Kerkhoffs in Foot & Ankle International

sj-docx-3-fai-10.1177_10711007251405240 – Supplemental material for Arthroscopic Bone Marrow Stimulation for Non-primary Osteochondral Lesions of the Talus Yields Limited Improvements in Patient-Reported Outcomes Compared to Primary Lesions: A Prospective 2-Year Follow-up Study Supplemental material, sj-docx-3-fai-10.1177_10711007251405240 for Arthroscopic Bone Marrow Stimulation for Non-primary Osteochondral Lesions of the Talus Yields Limited Improvements in Patient-Reported Outcomes Compared to Primary Lesions: A Prospective 2-Year Follow-up Study by Quinten G. H. Rikken, Jari Dahmen, Julian J. Hollander, Jason A. H. Steman, Sjoerd A. S. Stufkens and Gino M. M. J. Kerkhoffs in Foot & Ankle International

sj-docx-4-fai-10.1177_10711007251405240 – Supplemental material for Arthroscopic Bone Marrow Stimulation for Non-primary Osteochondral Lesions of the Talus Yields Limited Improvements in Patient-Reported Outcomes Compared to Primary Lesions: A Prospective 2-Year Follow-up Study Supplemental material, sj-docx-4-fai-10.1177_10711007251405240 for Arthroscopic Bone Marrow Stimulation for Non-primary Osteochondral Lesions of the Talus Yields Limited Improvements in Patient-Reported Outcomes Compared to Primary Lesions: A Prospective 2-Year Follow-up Study by Quinten G. H. Rikken, Jari Dahmen, Julian J. Hollander, Jason A. H. Steman, Sjoerd A. S. Stufkens and Gino M. M. J. Kerkhoffs in Foot & Ankle International

sj-docx-5-fai-10.1177_10711007251405240 – Supplemental material for Arthroscopic Bone Marrow Stimulation for Non-primary Osteochondral Lesions of the Talus Yields Limited Improvements in Patient-Reported Outcomes Compared to Primary Lesions: A Prospective 2-Year Follow-up Study Supplemental material, sj-docx-5-fai-10.1177_10711007251405240 for Arthroscopic Bone Marrow Stimulation for Non-primary Osteochondral Lesions of the Talus Yields Limited Improvements in Patient-Reported Outcomes Compared to Primary Lesions: A Prospective 2-Year Follow-up Study by Quinten G. H. Rikken, Jari Dahmen, Julian J. Hollander, Jason A. H. Steman, Sjoerd A. S. Stufkens and Gino M. M. J. Kerkhoffs in Foot & Ankle International

sj-docx-6-fai-10.1177_10711007251405240 – Supplemental material for Arthroscopic Bone Marrow Stimulation for Non-primary Osteochondral Lesions of the Talus Yields Limited Improvements in Patient-Reported Outcomes Compared to Primary Lesions: A Prospective 2-Year Follow-up Study Supplemental material, sj-docx-6-fai-10.1177_10711007251405240 for Arthroscopic Bone Marrow Stimulation for Non-primary Osteochondral Lesions of the Talus Yields Limited Improvements in Patient-Reported Outcomes Compared to Primary Lesions: A Prospective 2-Year Follow-up Study by Quinten G. H. Rikken, Jari Dahmen, Julian J. Hollander, Jason A. H. Steman, Sjoerd A. S. Stufkens and Gino M. M. J. Kerkhoffs in Foot & Ankle International

sj-docx-7-fai-10.1177_10711007251405240 – Supplemental material for Arthroscopic Bone Marrow Stimulation for Non-primary Osteochondral Lesions of the Talus Yields Limited Improvements in Patient-Reported Outcomes Compared to Primary Lesions: A Prospective 2-Year Follow-up Study Supplemental material, sj-docx-7-fai-10.1177_10711007251405240 for Arthroscopic Bone Marrow Stimulation for Non-primary Osteochondral Lesions of the Talus Yields Limited Improvements in Patient-Reported Outcomes Compared to Primary Lesions: A Prospective 2-Year Follow-up Study by Quinten G. H. Rikken, Jari Dahmen, Julian J. Hollander, Jason A. H. Steman, Sjoerd A. S. Stufkens and Gino M. M. J. Kerkhoffs in Foot & Ankle International
