# Supplementary material for: Arthroscopic Bone Marrow Stimulation for Non-primary Osteochondral Lesions of the Talus Yields Limited Improvements in Patient-Reported Outcomes Compared to Primary Lesions: A Prospective 2-Year Follow-up Study
Source: Foot Ankle Int. 2026 Feb 24;47(3):291–301. doi: 10.1177/10711007251405240 (PMC12966365; doi:10.1177/10711007251405240)
Supplement: sj-docx-1-fai-10.1177_10711007251405240 – Supplemental material for Arthroscopic Bone Marrow Stimulation for Non-primary Osteochondral Lesions of the Talus Yields Limited Improvements in Patient-Reported Outcomes Compared to Primary Lesions: A Prospective 2-Year Follow-up Study [file sj-docx-1-fai-10.1177_10711007251405240.docx]

# Appendix

**Appendix 1: Spearman’s Rho analysis for the Association of Continuous Baseline Variables with the Primary Outcome**

| **Variable** | **Primary Group (N= 25)** | **Non-Primary Group (N=17)** |
| --- | --- | --- |
| Age | ρ = 0.4  **P = 0.04** | ρ = 0.1  P= 0.72 |
| BMI | ρ = 0.3  P = 0.13 | ρ = 0.1  P = 0.62 |
| Lesion Size | ρ = -0.1  P = 0.70 | ρ = 0.1  P = 0.60 |
| Lesion Depth | ρ = -0.1  P = 0.60 | ρ = 0.3  P = 0.28 |

**Abbreviations** N= number of, BMI = body mass index, of note: 17 patients were assessed in the non-primary group as 2 out of 19 cases underwent revision and thus were not available.

**Appendix 2: Subgroup Comparison of the Primary Outcome (Change NRS during Weightbearing Pre- to Postoperatively)**

| **Variable** | **Primary Group**  **(N= 25)** |  | **Non-Primary Group (N=17)** |  |
| --- | --- | --- | --- | --- |
| ***2 Subgroups*** | Median (IQR) | P-Value | Median (IQR) | P-Value |
| *Sex*   - Male - Female | 3 (2 – 5)  4 (0 – 7) | 0.26 | 1.5 (0.5 – 3)  1 (1 – 1) | 0.41 |
| *BMS technique*   - Debridement only - Debridement with microfracturing | 0 (-1 – 1)  4 (1 – 5) | 0.56 | 1 (1 – 1)  1 (0.5 – 3) | 0.99 |
| *Prior Surgical Technique*   - BMS - Other* | N.a. | N.a. | 1 (1 – 3)  1 (-2 – 1) | 0.45 |
| *Concomitant Surgery*   - Yes - No | 8 (-1 – 8)  3 (1 – 5) | **0.03** | 0.5 (0 – 1)  1 (1 – 3) | 0.65 |
| ***>2 Subgroups*** |  |  |  |  |
| *Morphology*   - Cystic - Crater - Fragment | 5 (1 – 7)  3 (2 – 5)  3 (1 – 5) | 0.77 | 2.5 (1 – 3.5)  1 (0 – 3)  1 (1 – 1) | 0.43 |
| *OA Stage*   - Stage 0 - Stage 1 - Stage 2 - Stage 3 | 2.5 (1 – 4)  3.5 (1 – 5)  5 (5 – 10)  - | 0.16 | 2 (1 – 3)  1 (1 – 3)  0 (N.a.)  - | 0.42 |

**Abbreviations** N= number of, BMS= bone marrow stimulation, OA= osteoarthritis, N.a.= not applicable.

*Other group includes 1 case of prior fixation and 2 cases prior bone grafting. Of note: 17 patients were assessed in the non-primary group as 2 out of 19 cases underwent revision and thus were not available.

**Appendix 3: all other non-surgically related adverse events besides complications.**

| **Case** | **Age Sex** | **Procedure** | **Lesion / OA grade** | **Event** | **Pre- to postoperative change in primary outcome** |
| --- | --- | --- | --- | --- | --- |
| 1 | 23F | Primary BMS (MFx) | Area: 128,0mm^2^  Morphology: fragment  Zone: 4  OA grade: 1 | Received an intra-articular injection with corticosteroids 13 months postoperatively for symptomatic anterior synovitis. | Pre: 6  Post: 1  Change: 5 |
| 2 | 16F | Primary BMS (MFx) | Area: 132,7mm^2^  Morphology: crater  Zone: 4  OA grade: 0 | 4 weeks of soft cast with weightbearing as tolerated for soft-tissue impingement FHL/FDL tendons 12 months postoperatively. | Pre: 3  Post: 2  Change: 1 |
| 3 | 26F | Primary BMS (MFx) | Area: 23,7mm^2^  Morphology: crater  Zone: 9  OA grade: 0 | Traumatic Weber A distal fibula avulsion fracture after missing a step for which 2 weeks non-weightbearing softcast, 2 weeks weightbearing softcast, and 2 weeks walker. | Pre: 6  Post: 4  Change: 2 |
| 4 | 34M | Non-Primary BMS (MFx) | Area: 69,5mm^2^  Morphology: cyst  Zone: 4  OA grade: 2 | Received an intra-articular injection with corticosteroids 12 months postoperatively for symptomatic anterior synovitis. | Pre: 8  Post: 8  Change: 0 |
| 5 | 28M | Primary BMS (MFx) | Area: 86,9mm^2^  Morphology: crater  Zone: 4  OA grade: 0 | Received an intra-articular injection with corticosteroids 8 months postoperatively for symptomatic anterior synovitis. | Pre: 8  Post: 5  Change: 3 |

**Abbreviations:** F = Female, M = Male, BMS = Bone marrow stimulation, MFx = microfracturing

Of note: Lesion zone according to Raikin et al and ankle OA grading according to Cohen et al.
